# A comparison between dosages and plasma concentrations of dexmedetomidine in clinically ill patients: a prospective, observational, cohort study in Japan

**DOI:** 10.1186/2052-0492-1-15

**Published:** 2013-12-20

**Authors:** Yoshihito Fujita, Koichi Inoue, Tasuku Sakamoto, Saya Yoshizawa, Maiko Tomita, Yoshie Maeda, Hitomi Taka, Ai Muramatsu, Youichiro Hattori, Hiroyuki Hirate, Toshimasa Toyo’oka, Kazuya Sobue

**Affiliations:** Department of Anesthesiology and Medical Crisis Management, Nagoya City University Graduate School of Medical Sciences, Nagoya, 467-8601 Japan; Laboratory of Analytical and Bio-Analytical Chemistry, School of Pharmaceutical Sciences, University of Shizuoka, 52-1 Yada, Suruga-ku, Shizuoka, 422-8526 Japan

**Keywords:** Dexmedetomidine, Plasma concentration, Ultra performance liquid chromatography tandem mass spectrometry

## Abstract

**Background:**

Dexmedetomidine is a highly selective central α_2_-agonist with anesthetic and analgesic properties for patients in intensive care units. There is little information about the relationship between dosage and plasma concentration during long drug infusions of dexmedetomidine in critically ill patients, especially in Asians. In addition, the administration of dexmedetomidine with a dosage of 0.2–0.7 μg/kg/h in Japan is different from that with a dosage of 0.2–1.4 μg/kg/h in European countries and the USA. There has been concern about obtaining an effective concentration with a small dosage and estimating the relationship between dosage and plasma concentration. We conducted a prospective, observational, cohort study measuring plasma dexmedetomidine concentrations.

**Methods:**

Plasma dexmedetomidine concentrations of 67 samples from 34 patients in an intensive care unit for 2 months were measured by ultra performance liquid chromatography coupled with tandem mass spectrometry using single-blind method, and the correlation coefficient between dosages and plasma concentrations was estimated. Exclusion criteria included young patients (<16 years) and samples obtained from patients in which the dosage of dexmedetomidine was changed within 3 h.

**Results:**

Among the patients, 20 (58.8%) of the 34 received dexmedetomidine at 0.20–0.83 μg/kg/h, and in 40 of the 67 samples for which dexmedetomidine had been administered, this occurred for a median duration of 18.5 h (range, 3–87 h). The range of the dexmedetomidine plasma concentration was 0.22–2.50 ng/ml. By comparison with other studies, with a dosage of 0.2–0.7 μg/kg/h, the patients in this setting could obtain an effective dexmedetomidine concentration. The plasma dexmedetomidine concentration was moderately correlated with the administered dosage (*r* = 0.653, *P* < 0.01). The approximate linear equation was *y* = 0.171*x* + 0.254. The range of Richmond Agitation-Sedation Scale was 0 to -5.

**Conclusions:**

We concluded that, with a dosage of 0.2–0.83 μg/kg/h, the patients in this setting could obtain an effective dexmedetomidine concentration of 0.22–2.50 ng/ml. In addition, the plasma dexmedetomidine concentration was moderately correlated with the administered dosage (*r* = 0.653, *P* < 0.01).

**Trial registration:**

University Hospital Medical Information Network Clinical Trials Registry (UMIN-CTR) UMIN000009115.

## Background

Dexmedetomidine is a highly selective central α_2_-agonist with anesthetic and analgesic properties for patients in intensive care units (ICUs). In clinical settings, we administer dexmedetomidine in intensive care units at a dosage of 0.2–0.7 μg/kg/h because there is no commercially available blood concentration simulator. In Japan, a dosage of 0.2–0.7 μg/kg/h was used because medical insurance approved doses within this range. There is little information about the relationship between dosage and plasma concentration during long drug infusions of dexmedetomidine in critically ill patients [1, 2]. In addition, adaptation to Asian patients using data on the dexmedetomidine dosage and concentration from Caucasian patients may be a cause of concern because of racial differences. The purpose of this study was to confirm the effective dexmedetomidine concentration with a dosage of 0.2–0.7 μg/kg/h and to estimate the correlation coefficient between dosages and plasma concentrations in Japanese patients. We conducted a prospective, observational, cohort study measuring plasma dexmedetomidine in an intensive care unit by ultra performance liquid chromatography coupled with tandem mass spectrometry.

## Methods

This study (UMIN number 000009115) was conducted in the ICU of Nagoya City University Hospital, Japan. The study protocol was approved by our institutional ethics committee. Written informed consent was waived by the institutional ethics committee because the study was performed using residual blood (less than 1.0 ml) discarded from blood gas analysis in the patients. However, before analysis, we provided an oral description of the study and obtained agreement, which was documented in the medical record. This prospective, observational, cohort study involved the recruitment of patients sequentially admitted to the intensive care unit of Nagoya City University Hospital for 2 months. All patients admitted to the ICU were eligible, with the exception of patients less than 16 years old and patients in whom dosages of dexmedetomidine were changed within 3 h. Patients with a history of intolerance to dexmedetomidine or with significant metabolic, hematological, or endocrine disease were excluded.

Patients received continuous infusion of dexmedetomidine (Precedex®, Hospira Japan, Osaka, Japan) at 0.2–0.7 μg/kg/h without a loading dose. The dosage was administered on the basis of clinical need and adjusted as considered necessary to maintain optimal sedation. As concomitant treatment, patients received the standard care of the unit, which included administration of fentanyl or epidural anesthesia with 0.125% levobupivacaine for pain relief and midazolam and propofol for sedation. In the ICU, arterial blood gas analysis was performed routinely at 5–7 a.m. Residual blood (less than 1.0 ml) discarded from this blood gas analysis in the patients was used for this study. Arterial blood samples (less than 1.0 ml) from all the patients in the ICU during the survey period were collected in EDTA tubes immediately. Samples were kept at 4°C and centrifuged. Plasma was frozen at -80°C after separation and stored until analysis.

Measurement of the concentration of dexmedetomidine was performed using single-blind method. Arterial blood samples were numbered without information about the patients, even in terms of whether dexmedetomidine had been administered or not. In addition, these samples labeled by number were sent for measurement, and measurement was started without information about the patients. An analytical assay for the determination of dexmedetomidine was developed; the detailed description of which has been published previously [3]. Briefly, the quantification of dexmedetomidine was carried out using the original stable dexmedetomidine-*d*_3_ for electrospray ionization-tandem mass spectrometry. Efficacious concentration levels (50 pg/ml to 5 ng/ml) could be evaluated using a very small amount of plasma (10 μl). The lower limit of quantification was 5 pg/ml in the plasma.

Statistical analyses were performed with the computer program Statistical Package for the Social Sciences (SPSS 19.0, Chicago, IL, USA). Power analysis was calculated for the primary endpoint to estimate the correlation coefficient between dosages and plasma concentrations. We obtained preliminary data and estimated the correlation coefficient (*r*) to be approximately 0.5–0.6. A total sample size of 25–38 was required to detect a correlation coefficient of 0.5–0.6 assuming two-tailed type I error of 5% and type II error of 10%. Data are expressed as the mean ± standard deviation, median (interquartile range) for nonnormally distributed variables (Kolmogorov-Smirnov test), or number and percentage as appropriate. The correlation between dosages and plasma concentrations of dexmedetomidine was determined by linear regression. Correlation coefficient was calculated with Pearson's *r* or Spearman's *ρ* by the type of distribution. All *P* values are two-tailed. *P* values less than 0.01 were considered significant.

## Results and discussion

### Results

**Figure 1:**
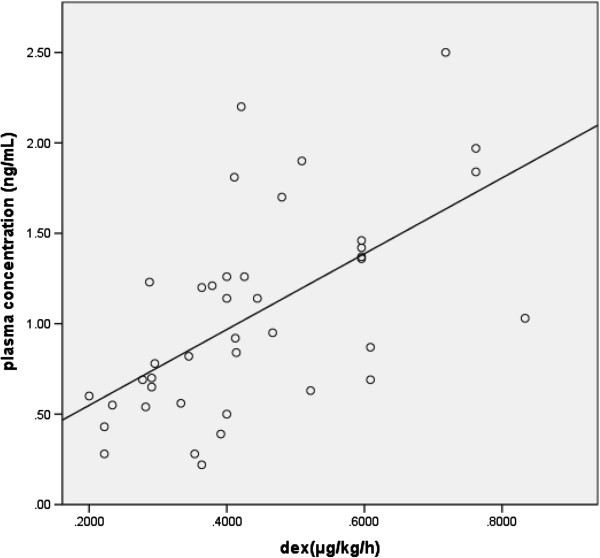
**The correlation coefficient between dosages and plasma concentrations.** The white circles and strait line indicated the result of each sample and the linear equation, respectively. The plasma dexmedetomidine concentration was moderately correlated with the administered dosage (*r* = 0.653, *P* < 0.01). The approximate linear equation was *y* = 0.171*x* + 0.259.

The characteristics of the patients are summarized in Table 1. Among the patients, 20 (58.8%) of the 34 received dexmedetomidine at 0.20–0.83 μg/kg/h, and in 40 samples for which dexmedetomidine had been administered, this occurred for a median duration of 18.5 h (range, 3–87 h). The five patients who required dexmedetomidine infusion for 87 h (more than 3.5 days) had three or four samples per patient. For nine patients, multiple samples were obtained (>1). The range of dexmedetomidine plasma concentration was 0.22–2.50 ng/ml (Table 2). The plasma dexmedetomidine concentration was moderately correlated with the administered dosage (*r* = 0.653, *P* < 0.01; Figure 1). The approximate linear equation was *y* = 0.171*x* + 0.259. The range of Richmond Agitation-Sedation Scale was 0 to -5 (Table 2). With a small dosage of 0.2–0.7 μg/kg/h, the patients in this setting could obtain an effective dexmedetomidine concentration. We confirmed that a concentration of 0 ng/ml dexmedetomidine was measured with the samples from the patients who were administered other sedative drugs including midazolam, fentanyl, propofol, oral diazepam, and oral clonidine hydrochloride without receiving dexmedetomidine.

**Table 1:** **Characteristics of the patients**

Characteristic	Value
Number	20
Age (year)	67.5 (41.5, 75.5)
Male, female	14, 6
Height (cm)	162.4 ±9.7
Weight (kg)	56.9 ±12.1
Main reason for ICU admission, number (%)	
Medical	7 (35 %)
Surgical	13 (65 %)

Values are mean ± SD or median (interquartile range) or number (%).

**Table 2:** **Details of administered dexmedetomidine and sedation**

Drug treatment	***N*** = 40
Duration of infusion (h)	18.5 (10, 46.25) [range 3–87]
Plasma concentrations (ng/ml)	1.05 ± 0.56 [range 0.22–2.50]
Dosages (μg/kg/h)	0.41 (0.30, 0.58) [range 0.20–0.83]
Combined administration	
No drug	14 (35 %)
1 drug	18 (45 %)
2 drugs	6 (15 %)
3 or more drugs	2 (5 %)
Fentanyl	23 (57.5 %)
Midazolam	3 (7.5 %)
Intubation	26 (65 %)
RASS	
≧1	0 (0 %)
0	16 (40 %)
-1	5 (12.5 %)
-2	6 (15.0 %)
-3	6 (15.0 %)
-4	4 (10.0 %)
-5	3 (7.5 %)

Values are mean ± SD or median (interquartile range) or number (%).

### Discussion

Our study demonstrated that, at a small dosage of 0.2–0.83 μg/kg/h, the Japanese patients in this clinical setting could obtain an effective dexmedetomidine concentration of 0.22–2.50 ng/ml. We also found that the plasma dexmedetomidine concentration was moderately correlated with the administered dosage. We thought that this information might be useful in a clinical setting for Japanese patients.

The effective sedative concentration of dexmedetomidine was thought to be between 0.2 and 3.2 ng/ml in previous studies. Ebert et al. described in detail the estimation of sedative effect and cold pressor test with several concentrations in ten healthy men (20–27 years old) [4]. In their study, recall and recognition decreased at a dose of more than 0.7 ng/ml. Pain response to the cold pressor test decreased at more than 3.2 ng/ml. This result suggests that a decrease of pain response might require more than 3.2 ng/ml; however, suppression of recognition might occur at 0.7 ng/ml. Despite not directly measuring dexmedetomidine concentration, Hall et al. reported sedative properties of a small dose of dexmedetomidine [5]. In their study, seven healthy volunteers received 0.2 or 0.6 μg/kg/h dexmedetomidine after a 10-min initial dose of 6 μg/kg/h. In this study, only 0.2 μg/kg/h dexmedetomidine suppressed the visual analog scale of pain. According to these papers, our dexmedetomidine range of 0.22–2.50 ng/ml might be within the effective sedative concentration.

At a dosage of 0.2–0.83 μg/kg/h, the Japanese patients in this clinical setting could obtain an effective dexmedetomidine concentration of 0.22–2.50 ng/ml; this data might be informative for Asian people. Adaptation to Asian patients using the data on dexmedetomidine dosage and concentration from Caucasian patients remains a cause of concern because of racial differences. These racial differences consist of not only differences in physique between Asians and Caucasians but also disparities in pain [6, 7]. Konstantatos et al. investigated the relationship between race and early opioid consumption. In this paper, they described that Chinese patients in Hong Kong required less opioid and experienced greater pain intensity and pruritus than Caucasian patients [6]. However, in our study at a range of 0.22–2.50 ng/ml, we could obtain an effective sedation level because the range of Richmond Agitation-Sedation Scale was 0 to -5. Our data demonstrated that the Japanese effective concentration range of dexmedetomidine might be almost equal to that of Caucasians.

The pharmacokinetics of dexmedetomidine in healthy volunteers [4, 5, 8, 9] might differ from that in intensive care patients [1, 2]. There is little information on its pharmacokinetics after long-term (>48 h) infusion in an intensive care unit setting [2]. Our samples were obtained in an ICU, and the median duration of our data was 18.5 h (range, 3–87 h). Our data might be useful and informative for long-term sedation, especially in Asians.

In this study, we administered continuous infusion of dexmedetomidine without a loading dose because many patients had already received other analgesic and sedative drugs, and we wanted to avoid abrupt hemodynamic effects [4] with a loading dose. In terms of the pharmacodynamic effect, the plasma concentration might vary because of the difference of infusion duration prior to blood sampling, even at a constant administration rate of dexmedetomidine. This study was an observational study, so we measured the blood samples in a clinical setting. In addition, we adopted the samples after continuous administration for more than 3 h because we could confirm an effect of administration of dexmedetomidine within 2–3 h after continuous administration in a clinical setting. With a loading dose, the correlation of the plasma dexmedetomidine concentration might be stronger with the administered dosage.

Our study had several other limitations. First, the number of patients was small, and the patient characteristics varied. Because this study was observational, as concomitant treatment, patients received the standard care of the unit, which included other pain relief and sedation drugs, such as the administration of fentanyl, epidural anesthesia, midazolam, and propofol. In future studies, by selecting patients undergoing a certain type of surgery, by controlling other pain relief and sedation drugs, as well as using estimated pain, we may be able to obtain more precise data concerning the strategy for administering dexmedetomidine. Second, we could not completely rule out that the measurement had been affected by the other drugs. We confirmed that a dexmedetomidine concentration of 0 ng/ml was measured in the samples from the patients who had been administered other sedative drugs including midazolam, fentanyl, propofol, oral diazepam, and oral clonidine hydrochloride without receiving dexmedetomidine. However, these findings could not guarantee that our method of measuring the dexmedetomidine concentration was unaffected by these other drugs. This might be considered a typical limitation in a clinical setting. Third, the five patients who required dexmedetomidine infusion for 87 h (more than 3.5 days) had three or four samples per patient. In total, multiple samples were obtained from nine patients (>1). Therefore, we could not rule out that these multiple samples might have induced variation in findings for all the patients.

## Conclusions

In conclusion, we demonstrated that, at a dosage of 0.2–0.83 μg/kg/h, the Japanese patients in this clinical setting could obtain an effective dexmedetomidine concentration of 0.22–2.50 ng/ml. In addition, the plasma dexmedetomidine concentration was moderately correlated with the administered dosage (*r* = 0.653, *P* < 0.01).

## Authors' information

YF is an MD and PhD and is Associate Professor at the Department of Anesthesiology and Medical Crisis Management, Nagoya City University Graduate School of Medical Sciences. KI is a PhD and an instructor at the Laboratory of Analytical and Bio-Analytical Chemistry, School of Pharmaceutical Sciences, University of Shizuoka. TS is a pharmacist and staff member at the Laboratory of Analytical and Bio-Analytical Chemistry, School of Pharmaceutical Sciences, University of Shizuoka. TT is a PhD and Professor at the Laboratory of Analytical and Bio-Analytical Chemistry, School of Pharmaceutical Sciences, University of Shizuoka. SY, MT, YM, HT, AM, YH, and HH are MDs and staff members at the Department of Anesthesiology and Medical Crisis Management, Nagoya City University Graduate School of Medical Sciences. KS is an MD, PhD, and Professor at the Department of Anesthesiology and Medical Crisis Management, Nagoya City University Graduate School of Medical Sciences.
